# Mechanical limitation of compression depth in a common CPR training manikin

**DOI:** 10.1186/s41077-026-00471-6

**Published:** 2026-11-06

**Authors:** Jonatan Appelt, Lena Jakob, Marco Freschi, Bernd Flamm, Ann-Kathrin Heissel, Lukas Fiebelkorn, Axel Schmutz, Julian Ganter

**Affiliations:** Department of Anesthesiology and Critical Care, Faculty of Medicine, Medical Center - University of Freiburg, Freiburg, 79106 Germany

**Keywords:** Emergency medicine, Resuscitation, Basic life support, Simulation

## Abstract

Current CPR guidelines recommend a chest compression depth of 5–6 cm. During an in-hospital training project using a widely adopted manikin (Resusci Anne, Laerdal Medical), integrated distance sensors consistently recorded compression depths below this target range.

Further mechanical evaluation showed that a depth of 6 cm is not physically achievable with this model; the maximum displacement did not exceed 5.7 cm, even when measurements included chest components as recommended by the manufacturer. Although limited to a single, widely used manikin, these findings highlight the need to reassess calibration and mechanical design to ensure alignment with guideline-based CPR training and accurate performance feedback.

## Background

**Fig. 1:**
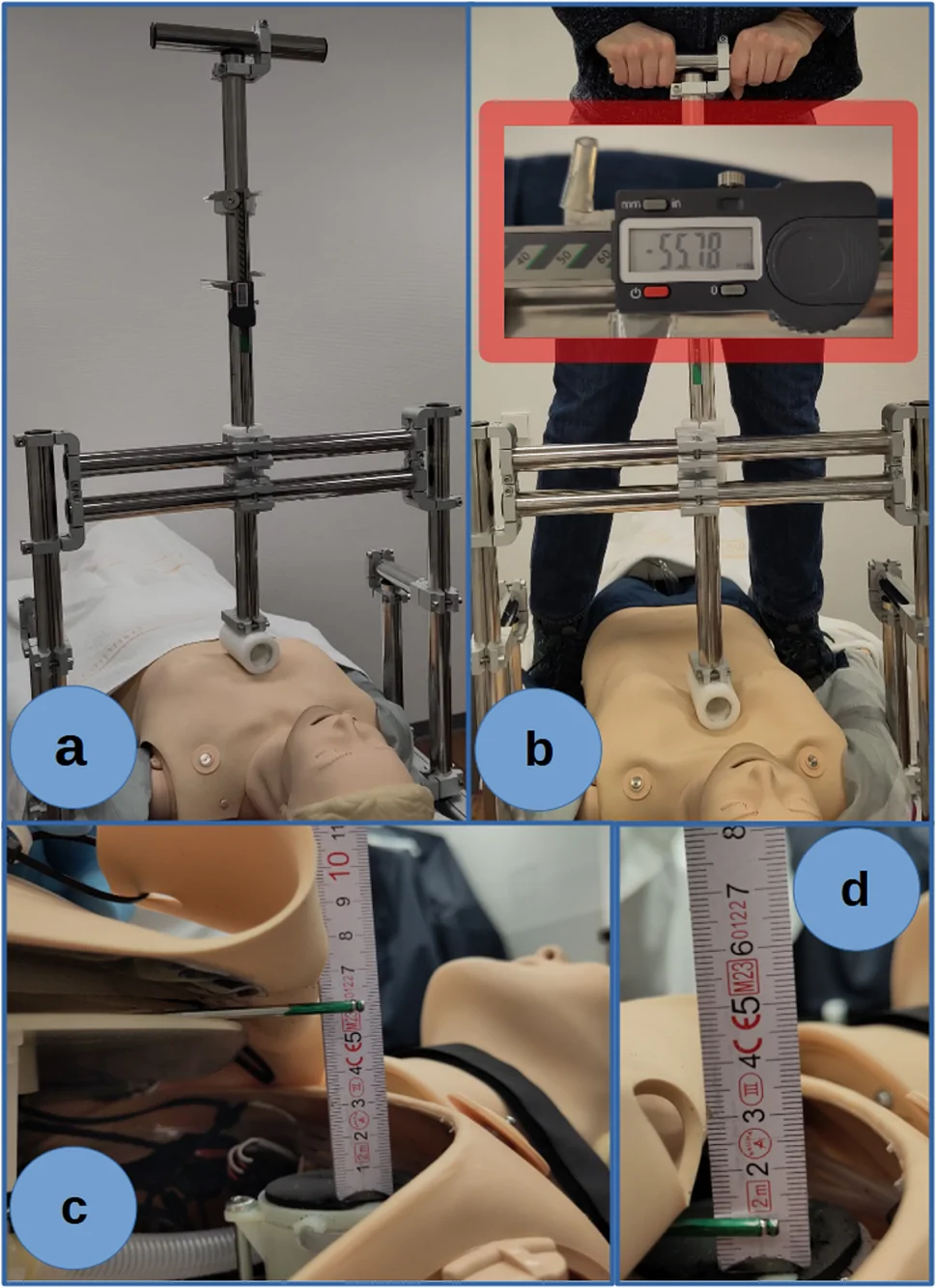
Manikin’s mechanical characteristics and limitations. **a**, **b** Representative images of a mechanical measuring device with linear guidance used for external measurement of indentation depth: **a** no compression and (**b**) maximum compression under an applied body weight of 75 kg. **c**, **d** Mechanical structure of the manikin after cranial retraction of the skin cover. **c** Chest recoil position, with the measuring scale aligned to the mechanical endpoint at 6 cm. d Maximum compression state, limited by the manikin’s structural design and not reaching the zero point on the scale

During a project on in-hospital resuscitation courses, we embedded distance sensors into standard Laerdal manikins (Resusci Anne Simulator; Laerdal Medical, Stavanger, Norway) -a widely used model for teaching CPR performance and recording compression depth parameters. However, we observed that the recorded compression depths were consistently below the target range of at least 5 cm, thus falling outside the recommended compression depth range of 5–6 cm in current resuscitation guidelines [1]. To further investigate this finding, we examined the mechanical properties of the entire manikin through external measurement and found that, although greater compression depths could be achieved overall, reaching a depth of 6 cm remained physically impossible due to mechanical limitations (Fig. 1).

### Investigation

The initial identification of this finding was performed using a high-precision Time-of-Flight distance sensor (Adafruit VL6180X) installed within the manikin, capturing the full displacement path. However, for the present analysis, an external measurement was performed using the setup shown in Fig. 1 to accurately determine the compression depth, including the skin and chest structures. External compression depth was defined as the vertical travel distance of the anterior chest surface.

All four available manikin models (including different generations) were assessed using the setup shown in Fig. 1. Each manikin was placed supine on a flat surface within a vertically guided metal frame, with height changes measured in millimeters using a caliper (Forits 150 mm, DIN 862). Measurements were performed in triplicate for each manikin. Compressions were measured at the external surface of the skin at the center of the thorax in accordance with guideline recommendations; minor cranial or caudal variations in the compression point did not affect the results. The load was applied vertically and centrally to the sternum. A body weight of 75 kg was sufficient to reach a clearly perceptible mechanical end point before full body weight was applied. This endpoint was defined as the stage at which no further increase in travel distance occurred despite the application force. The results are summarized in Table 1.

**Table 1:** Maximum achievable compression depth (cm) for each tested manikin model under a standardized load of 75 kg, including external measurements as illustrated in Fig. 1a. Measurements were performed in triplicate for each manikin. Values are presented as mean ± standard deviation (SD)

Manikin Model (Production Number/+Serial Number)	Maximum Compression Depth (cm)	Standard Deviation (cm)
Laerdal Resusci Anne Simulator (150-20033/15016130025)	5.6	0.05
Laerdal Resusci Anne Simulator (150-20033/15016130026)	5.7	0.02
Laerdal Resusci Anne Simulator (150-22000/15032233901)	5.8	0.06
Laerdal Resusci Anne Simulator (150-22000/15031233896)	5.8	0.03

### To discuss

**Fig. 2:**
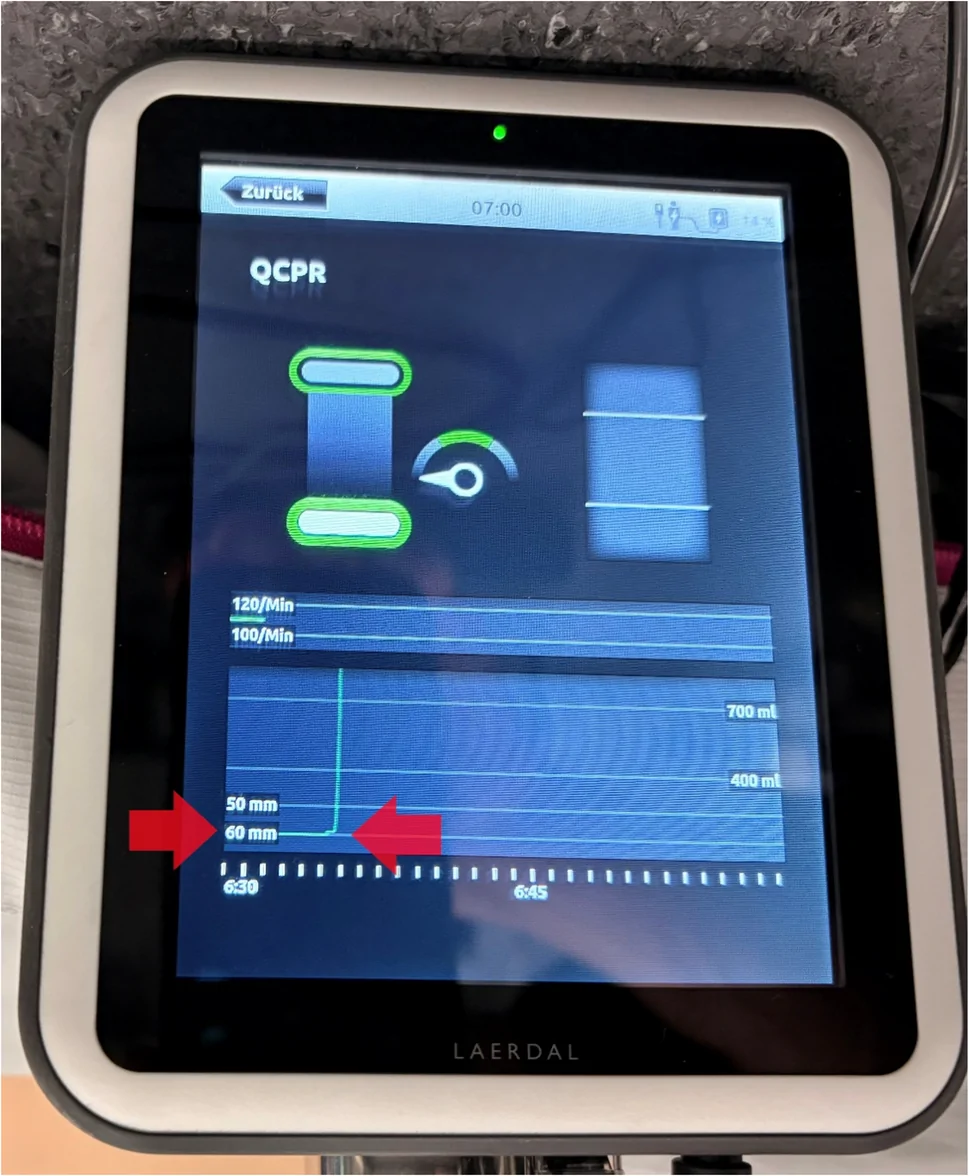
Display of the control unit (SimPad) at maximum compression of 60 mm (see arrows). The corresponding values are shown in millimeters (mm)

While the 2005 European Resuscitation Council (ERC) guidelines recommended compression depth of 4–5 cm, current guidelines specify a target depth of at least 5 cm but not more than 6 cm [1, 2]. Our findings indicated that achieving a depth greater than 5.7 cm is not mechanically feasible with these manikins. Despite following the manufacturer’s recommendation to include the skin and chest shields, a compression depth greater than 5.7 cm could not be achieved, highlighting a mechanical limitation of the manikin itself. Independently of this, an additional concern arises from the feedback system: how can manikin feedback systems display compressions as “too deep” when, mechanically, such depths cannot be achieved (Fig. 2)? Previous studies have shown that calibration discrepancies in CPR feedback devices can result in the displayed compression depth being overestimated [3]. In contrast, our measurements demonstrated stable and reproducible results that were largely independent of external factors, such as surface softness, consistent with findings reported in previous studies [4].

It is important to note that thoracic compressions involve dynamic force application, with transient peak forces potentially exceeding nominal force levels. Nevertheless, our findings indicate the presence of a mechanical resistance that could not be overcome, even under supramaximal loading conditions.

Furthermore, current manufacturer information states that the device is designed for “*chest compression (Guidelines 2015 depth)*” [5]. This corresponds to American Heart Association (AHA) guideline recommendations, which specify a chest compression depth of at least 5 cm [6]. Current resuscitation guidelines define the technical requirements that training devices must meet [7]. While manikin realism may vary [8], key parameters -such as compression depth- should be accurately reproduced. Accordingly, training devices and their successive generations should be adapted to remain consistent with evolving guideline recommendations.

Although the device technically fulfills guideline requirements by allowing compression depths beyond 5 cm, encountering a mechanical end point before reaching the upper recommended threshold may lead to a misleading learning effect, suggesting that effective compressions require exerting force against a mechanical limitation.

### Limitations

A key limitation of this observation is that, although the assessment was repeated multiple times, it was conducted using a manikin of a single type from a single manufacturer. However, this manikin represents the current model of a widely used product from a market-leading manufacturer.

## Conclusions

These findings warrant further investigation into the calibration and mechanical limits of training manikins. It is crucial that CPR training equipment accurately reflects guideline-based compression depths to ensure high-quality resuscitation education and reliable performance assessment.

## Data Availability

All data generated or analysed during this study are included in this published article.

## Abbreviations

AHA: American Heart Association
CPR: Cardiopulmonary Resuscitation
ERC: European Resuscitation Council

## References

[1] Smyth MA, Goor S van, Hansen CM, Fijačko N, Nakagawa NK, Raffay V, et al. European resuscitation council guidelines 2025 adult basic life support. Resuscitation. 2025;215. 10.1016/j.resuscitation.2025.110771. 41117574

[2] Handley AJ, Koster R, Monsieurs K, Perkins GD, Davies S, Bossaert L, European resuscitation council guidelines for resuscitation 2005. Section 2. Adult basic life support and use of automated external defibrillators. Resuscitation. 2005;67:S7–23. 10.1016/j.resuscitation.2005.10.007. 16321717

[3] Beesems SG, Koster RW. Accurate feedback of chest compression depth on a manikin on a soft surface with correction for total body displacement. Resuscitation. 2014;85:1439–43. 10.1016/j.resuscitation.2014.08.005. 25150184

[4] Yu BG, Oh JH, Kim Y, Kim TW. Accurate measurement of chest compression depth using impulse-radio ultra-wideband sensor on a mattress. PLoS ONE. 2017;12:e0183971. 10.1371/journal.pone.0183971. PMC5576690 28854262

[5] Laerdal Medical. Resusci Anne^®^ Simulator n.d. . Downloaded 2 June 2026.

[6] Kleinman ME, Brennan EE, Goldberger ZD, Swor RA, Terry M, Bobrow BJ, et al. Part 5: adult basic life support and cardiopulmonary resuscitation quality. Circulation. 2015;132:S414–35. 10.1161/CIR.0000000000000259. 26472993

[7] Nabecker S, de Raad T, Abelairas-Gomez C, Breckwoldt J, Chakroun-Walha O, Farquharson B, et al. European resuscitation council guidelines 2025 education for resuscitation. Resuscitation. 2025;215. 10.1016/j.resuscitation.2025.110739. 41117567

[8] Purkarthofer D, Weldi M, Trathnigg G, Billig S. Ventilation characteristics of cardiopulmonary resuscitation manikins: a mixed-methods bench study. Resusc Plus. 2026;29:101318. 10.1016/j.resplu.2026.101318. PMC13127201 42064543

